# A targeted mass spectrometry method for the accurate label-free quantification of immunogenic gluten peptides produced during simulated digestion of food matrices

**DOI:** 10.1016/j.mex.2020.101076

**Published:** 2020-09-25

**Authors:** Olivia Ogilvie, Nigel Larsen, Kevin Sutton, Laura Domigan, Juliet Gerrard, Nicholas Demarais, Sarah Roberts

**Affiliations:** a School of Biological Sciences, The University of Auckland, Private Bag 92019, Auckland 1142, New Zealand; b Riddet Institute, Massey University, Private Bag 11 222, Palmerston North 4442, New Zealand; c The New Zealand Institute for Plant & Food Research Limited, Private Bag 4704, Christchurch Mail Centre, Christchurch 8140, New Zealand; d Department of Chemical and Materials Engineering, The University of Auckland, Private Bag 92019, Auckland 1142, New Zealand; e School of Chemical Sciences, The University of Auckland, Private Bag 92019, Auckland 1142, New Zealand

**Keywords:** Peptidomics, Celiac disease, Bread, *In vitro* digestion, Wheat

## Abstract

Mass spectrometry (MS) is an emerging method to determine the accurate concentration of immunogenic gluten peptides. It is of interest to quantify specific peptides within the gluten peptidome due to the role they play in the activation of the celiac immune cascade. Celiac disease is an autoimmune disorder triggered in genetically susceptible individuals by the presence of specific gluten peptides that resist digestion in the gastrointestinal tract. The protocol detailed within this paper can accurately quantify (label-free) the concentration of six immunogenic gluten peptides (including the 33mer) released from a food matrix using the INFOGEST *in vitro* digestion protocol. This method can be used to monitor small changes in the concentration of these marker peptides in response to exogenous factors such as plant-breeding, fermentation or food processing.

## Graphical abstract

**Figure:**
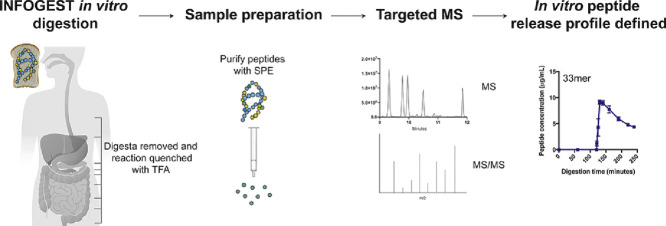

*Customisation*:

• A sample preparation method that allows the user to detect immunogenic gluten peptides within a food matrix digested using the INFOGEST *in vitro* digestion protocol with minimal matrix effects;
• A targeted, accurate, label-free mass spectrometry quantification method for six marker gluten peptides using an external standard curve that is compatible with the INFOGEST *in vitro* digestion;
• A methodology that can be expanded to include additional peptide targets such as other immunogenic gluten peptides or celiac peptides from other grains.

Specifications Table

**Table:** 

Subject Area	Biochemistry, Genetics and Molecular Biology
More specific subject area	Mass spectrometry of wheat gluten peptides
Method name	Targeted mass spectrometry method for gluten peptides
Name and reference of original method	van den Broeck, H. C., Cordewener, J. H. G., Nessen, M. A., America, A. H. P. Van der Meer, I. M. Label free targeted detection and quantification of celiac disease immunogenic epitopes by mass spectrometry. Journal of Chromatography A, Volume 1391, 24 April 2015, Pages 60–71. doi:10.1016/j.chroma.2015.02.070
Resource availability	

## Background

Celiac disease is an autoimmune disease initiated in genetically susceptible individuals by immunogenic gluten peptides that are resistant to gastrointestinal digestion [1]. Immunogenic gluten peptides range between 9 and 33 amino acids in length and possess epitopes that bind to the human leukocyte antigen (HLA)-DQ2 or 8 receptors [2]. Hundreds of gluten derived peptides have been identified that activate the celiac response. These peptides exhibit a hierarchy of immunodominance [3], meaning that some initiate a stronger immune response than others. Most often, immunogenic peptides are detected and validated within an isolated protein system that was digested using trypsin and/or chymotrypsin. This is notable because the digestion of gluten is altered by the presence of a food matrix [4]. Furthermore *in vivo*, immunogenic peptides are the product of pepsin, trypsin and chymotrypsin digestion.

To overcome these limitations, the protocol herein describes an accurate label-free mass spectrometry (MS) method to quantify six immunogenic gluten peptides (Table 1) within food matrices that have been digested using the INFOGEST *in vitro* assay [5], which aims to stimulate digestion within the gastrointestinal tract. This accurate MS method is built on a previous MS method described by van den Broeck 2015 [6] that was developed to determine the relative concentration of peptides P1-P6 within isolated protein systems. All six marker peptides contain at least one celiac epitope, are proline-rich and are unequivocally involved in activation of the celiac immune cascade (Table 1).

**Table 1:** The marker immunogenic gluten peptides analysed by the targeted PRM MS method described. All peptides are proline rich and contain at least one epitope that activates celiac pathogenesis.

Peptide name	Percent proline	Core epitope(s)	Amino acid sequence
P1	30.8	DQ2.5-glia-α1a (x1)	LQLQPFPQPQLPY
P2	30.8	DQ2.5-glia-α1a (x1) DQ2.5-glia-α2 (x1)	LQLQPFPQPQLPYPQPQPF
P3	38.5	DQ2.5-glia-α1a (x1) DQ2.5-glia-α2 (x1)	LQLQPFPQPQLPYPQPHLPYPQPQPF
P4	38.5	DQ2.5-glia-α1a (x1) DQ2.5-glia-α1b (x1) DQ2.5-glia-α2 (x2)	LQLQPFPQPQLPYPQPQLPYPQPQPF
33mer (P5)	39.4	DQ2.5-glia-α1a (x1) DQ2.5-glia-α1b (x2) DQ2.5-glia-α2 (x3)	LQLQPFPQPQLPYPQPQLPYPQPQLPYPQPQPF
P6	38.5	DQ2.5-glia-α3 (x1)	RPQQPYPQPQPQY

This manuscript is divided into three sections covering the sample digestion, sample preparation and the quantitative parallel reaction monitoring (PRM) MS method. Each section is further divided into the protocol, and the method development workflow used to develop that protocol. The method development workflow was included to allow the addition of alternative peptides if desired. The peptides investigated herein are α-gliadin derived; however, immunogenic gluten peptides have been identified from all gluten protein classes and therefore future users may want to include a wider range. Overall, this protocol can be applied to determine the accurate concentration of six immunogenic peptides, which are displayed in Table 1, within the digesta of food. When used at different points throughout digestion, the results can create a model of peptide release in response to different experimental and processing treatments.

## Materials

### Reagents

The following are specialised reagents required to undertake this protocol. In addition to these reagents, the user must obtain those required for the enzyme activity assays as detailed in the references below.

• Marker peptides P1-P6 in lyophilised form, 0.5 mg quantities at >98% purity (New England Peptide (MA, USA)).
• Methanol (AnalaR grade or better)
• Acetonitrile (ACN) (LC-MS grade)
• Formic acid (FA) (LC-MS grade)
• KCl
• KH_2_PO_4_
• NaHCO_3_
• NaCl
• MgCl_2_.(H_2_O)_6_
• (NH_4_)_2_CO_3_
• CaCl_2_
• Salivary α-amylase (Sigma: A6255)
• Pepsin (Sigma: P7012)
• α-chymotrypsin (Sigma: C4129)
• trypsin (Sigma: T0303)
• Trifluoroacetic acid (TFA) (LC-MS grade)

### Equipment

The following specialist equipment is required to undertake this protocol. General lab equipment is also required including pipettes and a centrifuge capable of spinning 1.5 mL microcentrifuge tubes at 14,000 × g.

• Orbitrap Q Exactive™ Plus (Thermo Fisher Scientific) (or equivalent)
• Vanquish ultra-high-performance liquid chromatography (UPLC) system (or equivalent)
• Aeris™ 1.7 µm PEPTIDE XB-C18 100 Å, LC Column 150 × 2.1 mm (Phenomenex) fitted with a C18-Peptide SecurityGuard™ ULTRA Cartridge (Phenomenex)
• Strata™-XL 100 µm Polymeric Reversed Phase, 30 mg/mL tubes (Phenomenex)
• Vacuum manifold
• SINGLE StEP™ filter vial (Thomson Instrument Company™) (or equivalent)

### Software

• Xcalibur^Ⓡ^ Suite (Thermo Fisher Scientific) (or equivalent)
• Microsoft^Ⓡ^ Excel^Ⓡ^ (or equivalent)

## Method details

### Sample digestion

This section describes the INFOGEST *in vitro* digestion assay [5] with minor modifications that make it compatible with the PRM MS method described below. The INFOGEST *in vitro* digestion assay was chosen as it simulates protein digestion in the human gastrointestinal tract [7]. The specific modification herein is the reaction quenching method. As well as the digestion protocol, the workflow employed to optimise the sample quenching method is described. Matrix effects occurred when pancreatin was used as the digestive component in the intestinal phase; therefore, the use of pure trypsin and chymotrypsin is recommended. This method is specifically optimised for the digestion of bread.

#### Method development workflow

Gluten derived peptides that activate celiac disease are rich in proline and glutamine residues and thus behave unusually in aqueous solution [8]. The method development workflow for the *in vitro* digestion protocol was centred on identifying an appropriate quenching reagent. Some quenching reagents can cause peptide precipitation and matrix effects—these are peptide-specific and unpredictable meaning they must be experimentally investigated.

During method development, various quenching reagents were selected from literature and their suitability assessed, including the addition of 70% ethanol at 1:1 v/v [9], 0.1% v/v TFA [10], ACN with 0.1% TFA at 1:1 v/v [11], no quenching [12], and an trypsin-chymotrypsin inhibitor from Glycine max (soybean) [5]. The compatibility of each quenching reagent was assessed by determining the immunogenic peptide abundance *via* PRM MS with and without the reagent. This experiment was undertaken in both a blank matrix and the sample matrix of bread digesta by spiking synthetic marker peptides at known concentrations. Only P1H was used in the sample matrix. If the presence of the quenching reagent decreased the marker peptide abundance it was not selected as the quenching reagent. The most effective quenching reagent for P1-P6 was 0.1% v/v TFA.

#### Final protocol

The enzymatic activity of α-amylase, pepsin, trypsin and chymotrypsin were defined as described in the supplementary material of the INFOGEST protocol [5]. The activity of α-amylase was determined using a spectrophotometric stop assay adapted from Bernfeld [13] by measuring the concentration of liberated maltose. The activity of pepsin was determined by a spectrophotometric stop assay adapted from Anson [14] using haemoglobin as a substrate. The activity of trypsin was established by a continuous spectrophotometric rate determination assay adapted from Hummel [15] using the substrate p-toluene-sulfonyl-L-arginine methyl ester (TAME). The activity of chymotrypsin was defined by continuous spectrophotometric rate determination assay adapted from Hummel [15] using the substrate N-Benzoyl-L-Tyrosine Ethyl Ester (BTEE). Bread was then digested in biological triplicate using the INFOGEST assay as follows:

1. Bread was freeze-dried.
2. Stock solutions of digestive fluids were formulated at 1.25 times the working concentration (Table 2) and stored at −20 °C for up to one year.

**Table 2:** The composition of the stock digestive fluids x1.25 working concentration.

Constituent	Oral (mmol L^−1^)	Gastric (mmol L^−1^)	Intestinal (mmol L^−1^)
KCl	15.1	6.90	6.80
KH_2_PO_4_	3.70	0.90	0.80
NaHCO_3_	13.6	25.0	85.0
NaCl	–	47.2	38.4
MgCl_2_.(H_2_O)_6_	0.15	0.10	0.33
(NH_4_)_2_CO_3_	0.06	0.50	–
pH	7.00	3.00	7.00
3. Simulated digestive fluids (Table 3) were prepared using the stock solutions adding the enzymatic components by their pre-defined activity.
  i. Fluids were prepared 25 min before required and prewarmed to 37 °C. For example, the intestinal fluid was prepared after 95 min of gastric digestion.
  ii. Enough fluid for all replicates was prepared in a stock solution to improve the method's reproducibility.

**Table 3:** The composition of the simulated digestion fluids used during the INFOGEST digestion assay. Enzymes were added based on predefined activity.

Oral	Gastric	Intestinal
SSF x1	SGF x1	SIF x1
α-amylase, 75 U mL^−1^	Pepsin, 2000 U mL^−1^	α-chymotrpysin, 25 U mL^−1^ and trypsin 100 U mL^−1^
CaCl_2_, 0.75 mmol L^−1^	CaCl_2_, 0.075 mmol L^−1^	CaCl_2_, 0.3 mmol L^−1^
milliQ water	milliQ water	milliQ water
pH 7	pH 3	pH 7
4. The freeze-dried sample was rehydrated with 1.25:4 v/w with milliQ water.
  i. For example, 1.25 g of freeze-dried bread was added to 4 mL of water.
  ii. If feasible (dependent on the sample volume), add an additional isotopically labelled internal standard at this step.
5. After rehydration, oral fluid was immediately added at a 1:1 v/w ratio with the original bread weight, and the sample was triturated with a fork to simulate chewing for 2 min
  i. For example, 1.25 mL of oral fluid was added to 1.25 g of bread.
6. Gastric fluid was added at a 1:1 v/w ratio with the total solution and incubated for 2 h at 37 °C whilst shaking.
  i. For example, 2.5 mL of gastric fluid was added to 1.25 g of bread.
7. The intestinal fluid was added at a 1:1 v/w ratio with the total solution and digested for 2 h at 37 °C, whilst shaking.
  i. For example, 5 mL of intestinal fluid was added to 1.25 g of bread.
  ii. If samples were removed during the gastric phase, this loss of volume was taken into account when the intestinal fluid was added.
8. At the desired time points, aliquots of digesta were removed and the reaction quenched with TFA from a 12% v/v stock to achieve 0.1% v/v.
  i. After TFA addition, samples were vortexed for 5 s, immediately snap-frozen in N_2(l)_, then stored at −20 °C for up to one year.
  ii. To efficiently quench the reaction, the appropriate volume of TFA was added to all sampling tubes at the start of the experiment.

### Sample preparation

Before MS, bread samples digested with the INFOGEST *in vitro* assay were prepared by solid-phase extraction (SPE) using Strata™-XL 100 µm Polymeric Reversed Phase, 30 mg mL^−1^ tubes (Phenomenex). Both experimental variability and matrix effects were monitored by addition of an isotopically labelled internal standard (P1H; Table 10) to the sample supernatant at 3 µg mL^−1^ before SPE.

#### Method development workflow

**Fig. 1:**
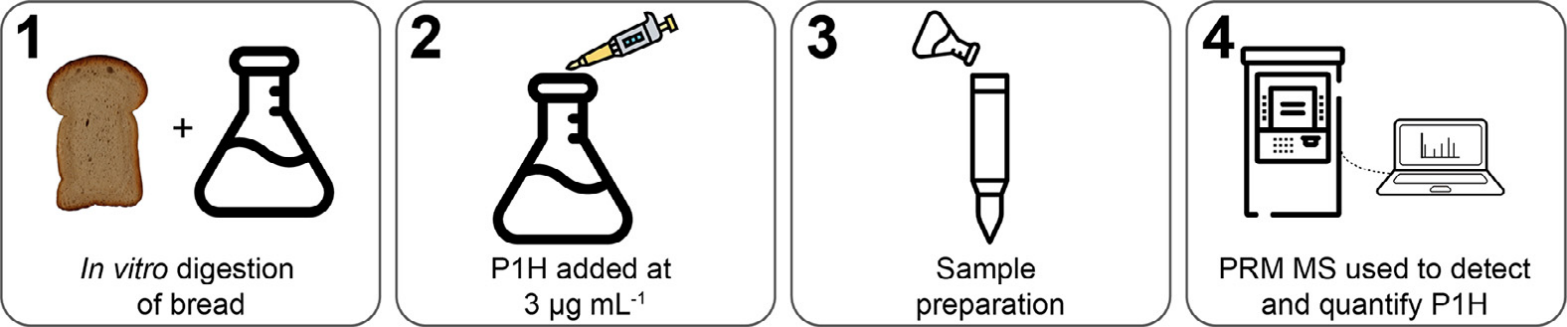
Workflow to screen the efficiency of the sample preparation method for new gluten derived peptides. Substitute P1H for new peptides.

There is no one-size-fits-all sample preparation method. Development of the SPE sample preparation method was centred on screening the efficiency of elution for various eluates using P1H and the workflow depicted in Fig. 1. SPE was chosen as the sample preparation method due to its predefined ability to reduce matrix effects and reduced sample handling time compared to other techniques [6,^10^,[16], [17], [18].

Briefly, bread was digested using the INFOGEST assay, reaction quenched, then the supernatant obtained by centrifugation. The isotopically labelled peptide P1H was then spiked into the supernatant at 3 µg mL^−1^ and digesta purified by SPE. The general SPE protocol detailed by the manufacturer (displayed below in Table 4) was employed substituting different eluates in *Step 5*. The eluates trialled were 50% MeOH, 50% ACN; 100% ACN, 1% formic acid; 100% MeOH; 79.5% ACN, 1% formic acid. Following peptide elution, the abundance of P1H was determined using PRM MS comparing the abundance within the cleaned sample matrix, to P1H dissolved in a blank control matrix. Once the most efficient eluate was identified, its efficacy was confirmed for peptides P2-P6 (non-isotopically labelled) in a blank sample matrix.

**Table 4:** SPE purification protocol.

Step number	Step name	Solution	Volume
1	Wash 1	100% methanol	1 mL x3
2	Wash 2	H_2_O/H^+^, pH 3	1 mL x2
3	Load	Sample	200 µL
4	Wash 3	5% ACN	1 mL x2
5	Elute	79.5% ACN, 1% FA	210 µL x2

#### Final protocol

1. Digesta was defrosted on ice, vortexed, then centrifuged at 14,000 × *g* for 10 min.
2. An aliquot of the supernatant was removed (200 µL) and P1H added from a 50 µg mL^−1^ stock to a final concentration of 3 µg mL^−1^.
3. Peptides were extracted by SPE from the digesta supernatant as detailed in Table 4.
  i. The SPE tubes were dried for at least 30 s between each step.
4. The eluate of Step 5 (Table 4) was collected and filtered using a SINGLE StEP™ filter vial (Thomson Instrument Company™),
  i. These samples were stored at 4 °C for 2–3 days or −20 °C for extended periods of time.

### Targeted mass spectrometry

The MS method herein was optimised using an Orbitrap Q Exactive™ Plus in PRM mode for the six marker immunogenic peptides displayed in Table 1, all of which are unequivocally involved in celiac disease.

#### Method development workflow

Peptides P1-P6 were selected from literature [6] and lyophilised synthetic versions were obtained in 0.5 mg quantitates (98% purity, New England Peptide). A PRM instrument method was assembled in Xcalibur, matrix effects assessed, the methods replicability determined and the limit of detection (LOD) and limit of quantification (LOQ) defined.

#### Assembling the PRM method

Each peptide was analysed individually in full scan data-dependent analysis (DDA) tandem fragmentation (MS/MS) mode using the ionisation and fragmentation parameters described in Table 5. Each peptide's retention time, dominant charge state and mass-to-charge ratio (*m/z*) were defined by examining the raw spectra. *In silico* MS/MS was undertaken using the PROTEOMICS TOOLKIT fragment ion calculator (or equivalent software). Direct sequence fragment ions (*b*^+^ and *y*^+^) were identified from the raw spectra by matching the *in silico* MS/MS ions to those experimentally observed. The highest intensity 4–5 direct sequence fragment ions were selected as identifier and quantifier ions for use in data processing. Using this information, a PRM method was assembled in the ‘Thermo Xcalibur Instrument Setup’ window.

**Table 5:** Instrument parameters for MS of gluten peptides on an Orbitrap Q Exactive^TM^ Plus.

Parameter	Value
Spray voltage	35 kV
Spray current	17 µA
Aux gas flow rate	10
Sheath gas flow rate	45
Scan range	108–4015 *m/z*
Resolving power	70,000
Capillary temperature	320 °C
Normalised collision energy	Stepped 18–27

#### Assessing matrix effects

**Fig. 2:**
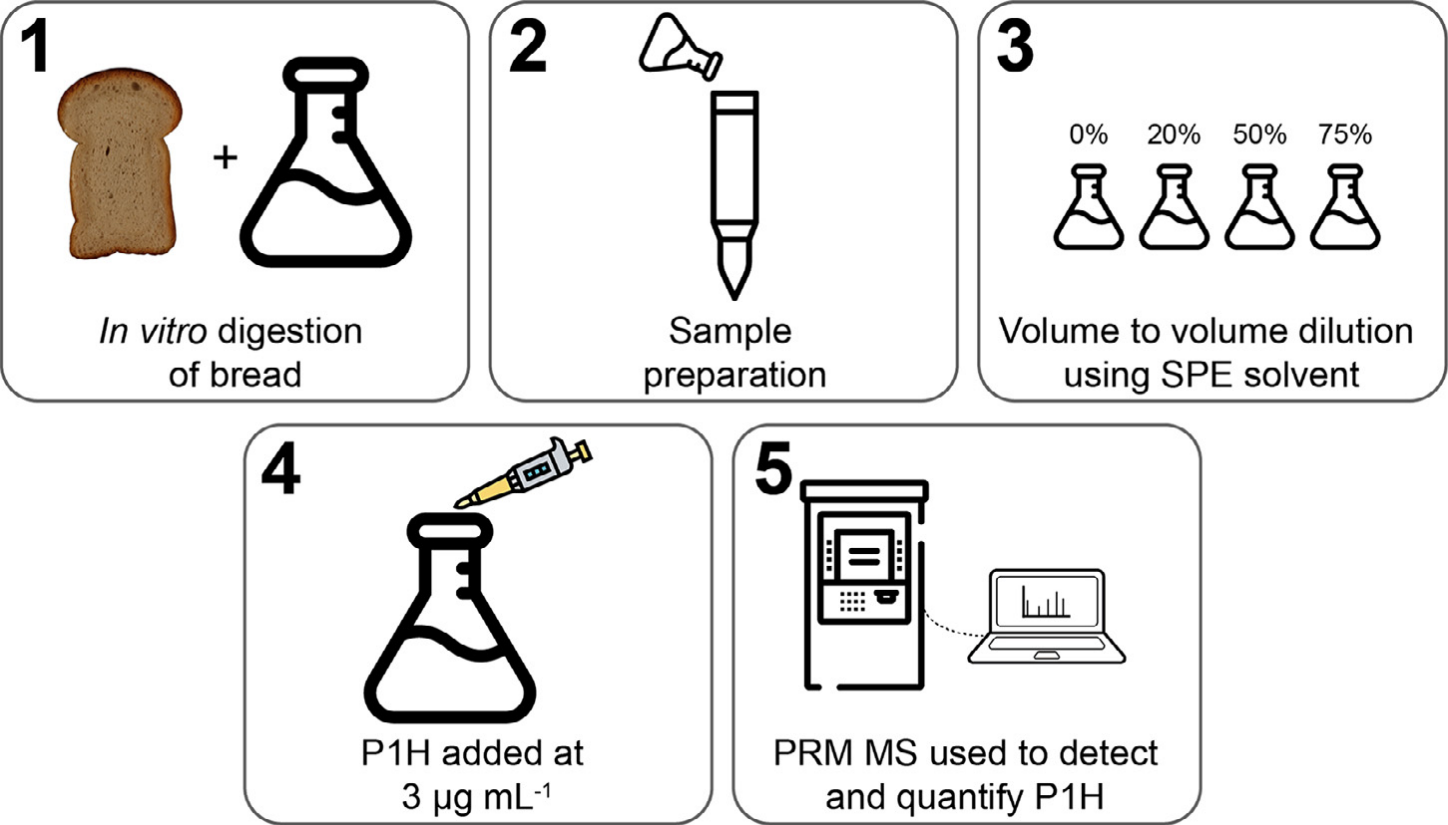
Workflow used to assess matrix effects during method development. Step 1 DIGEdigest the bread sample was digested and prepared *via* SPE (Step 2) without P1H. In step 3, the eluate was diluted to three concentrations, following which P1H was added to each (Step 4). The degree of matrix effects was then determined by comparing the peptide concentration within each matrix dilution by MS with a pure sample of P1H at 3 µg mL^−1^.

Matrix effects can occur during electrospray ionisation (ESI) due to the intrinsic competition for charge-transfer that occurs between analytes within the same aerosol droplet [19,20]. Matrix effects cause an analyte to be selectively suppressed or enhanced, which may unpredictably alter the peptide abundance and concentration calculated during PRM MS. An in-depth discussion of matrix effects and how to overcome them is detailed in Taylor 2005 [21]. Fig. 2 displays the workflow used to assess matrix effects due to components within the sample. The heavy (H) label indicates the isotopically labelled peptide.

Bread was digested using the INFOGEST assay, then prepared using the SPE as detailed above (Fig. 2). The SPE eluate was diluted serially to 75%, 50% and 20% in pure SPE eluate. The isotopically labelled peptide P1H was then added to each sample at 3 µg mL^−1^ and the peptide abundance was determined using PRM MS. The control sample contained 100% SPE eluate with P1H at 3 μg mL^−1^. The difference in P1H abundance between the pure and diluted samples represents the effect of the sample matrix.

#### Defining the methods replicability

The day-to-day replicability of the PRM and sample preparation method was defined by determining the peptide concentration within the same sample, over multiple days. To do so, *in vitro* bread digesta was separated into aliquots and frozen. Each day a sample was prepared (using the SPE protocol) and the abundance of P1-P6 determined using PRM MS. The values obtained were compared over subsequent days and coefficient of variation (CV) calculated using GraphPad. A CV of <10% indicates low day-to-day variability.

#### Defining the methods LOD and LOQ

The limit of detection (LOD) and limit of quantitation (LOQ) must be determined experimentally as they are unpredictable by regression modelling. The LOD is the lowest analyte concentration distinguishable from zero, and LOQ the lowest concentration that can be determined with quantitative accuracy [22]. The LOD and LOQ were defined for peptides P1-P6 using the method described by the International Council for Harmonisation of Technical Requirements^1^ (ICH) using ‘the Standard Deviation of the Response and the Slope’ .

Peptide P1-P6 were spiked (in triplicate) into a blank sample matrix previously purified by SPE (matrix matched). The sample was then diluted using SPE eluate at decreasing concentrations towards the limit of the assay, specifically to 10, 5, 2, 1, 0.5, 0.3, 0.1, 0.05, 0.03 and 0.01 µg mL^−1^. Each individual sample was injected and analysed using PRM MS at least five times [22]. The abundance (extracted ion current, defined using ‘Xcalibur Quant’) of each peptide was determined at each decreasing concentration; the LOQ was calculated using the standard deviation of the residuals as detailed in Eqs. (1) and (2) (for help with these calculations see https://www.youtube.com/watch?v=DXiGL72twow), and the LOD calculated using the standard deviation of the response and the slope as detailed in Eqs. (3) and (4) (for help with these calculations see https://www.youtube.com/watch?v=u7LCGkFuUFE).

(1) $$S=\;\sqrt{\frac{\sum \left(\gamma -\overset{⌢}{y}\right){\;}^{2}}{n-2}}$$

*S* = standard deviation of the residuals.

*n* = number of data points.

*γ* = residual values.

$\hat{y}$ = mean of the data values.

(2) $$LoQ=\;\frac{10\;\times S}{Slope}$$

*S* = standard deviation of the residuals.

*Slope* = obtained by linear regression.

(3) $$LoD=\;\frac{3.3\;\times S}{Slope}$$

*S* = standard deviation of the residuals.

*Slope* = obtained by linear regression.

(4) $$LoD=\bar{Y}+k\times S\left(Y\right)$$

$\hat{y}$ = mean value.

*k* = multiplication coefficient (10 [22]).

*S* = standard deviation of the mean.

#### Protocol for quantitative mass spectrometry

**Fig. 3:**
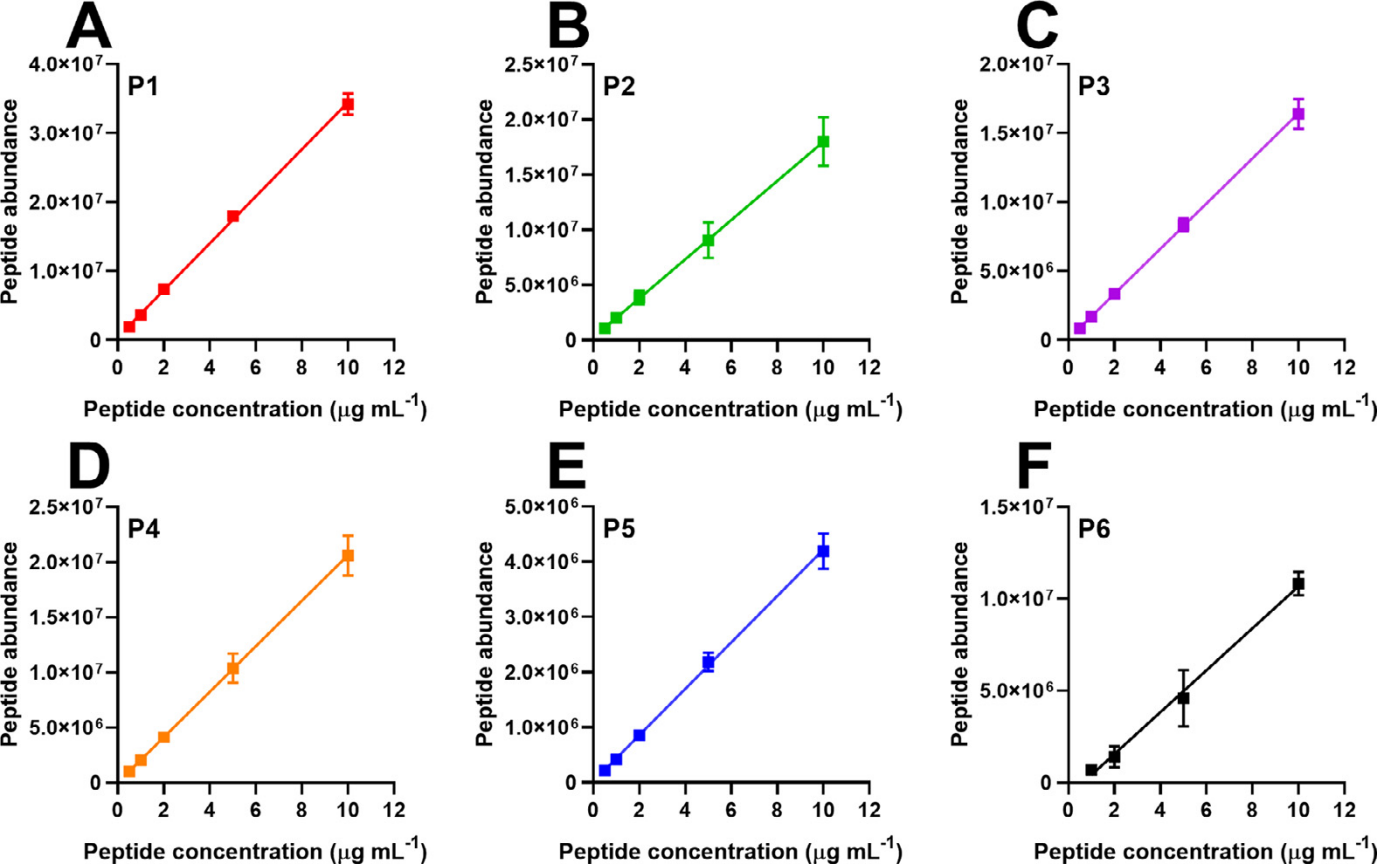
Peptides P1-P6 demonstrate a linear relationship between peptide abundance and peptide concentration between 0.5 and 10 µg mL^−1^ allowing the use of linear regression to predict the concentration of peptides with known abundance. (A) P1, (B) P2, (C) P3, (D) P4, (E) P5, and (F) P6. At least seven replicates were analysed. Error bars display the standard deviation of the mean.

This optimised PRM and sample preparation method can accurately quantify peptides P1-P6 with an LOD between 0.027–0.161 µg mL^−1^ (peptide dependent) and LOQ between 0.218–0.769 (Table 6). As highlighted in Fig. 3, P1-P6 display a linear relationship between peptide concentration and peptide abundance between 0.5 µg mL^−1^ and 10 µg mL^−1^, which extends to the defined LOQ. As recommended by the ICH^1^, Table 7 displays the typical regression properties of the calibration curves used to extrapolate the peptide abundance to concentration. The PRM method has minimal day-to-day variability, which is highlighted in Table 8 by a CV of <10%. Additionally, P1-P6 do not show matrix effects due to the efficiency of the SPE sample preparation method described above. This accurate MS method builds on the relative MS method developed by van den Broeck 2005 [6]. The optimised protocol is detailed in full:

1. A PRM method was assembled in ‘Thermo Xcalibur instrument setup’ using the transitions in Table 10 for the inclusion list.
  i. The chromatography schedule in Table 9 was added to this method, as was the MS instrument parameters in Table 5 above.

**Table 9:** Chromatography schedule used for quantitative mass spectrometry of gluten.

Elution time (minutes)	Percent of mobile phase B	Flow rate (mL min^−1^)
0	Inject sample	
1.00	7	0.30
3.00	25	0.30
3.90	40	0.30
8.00	95	0.40
9.50	95	0.40
12.0	95	0.30
12.5	7	0.30
15.0	7	End
2. An external standard curve was prepared using synthetic versions of P1-P6 between 0.5 and 10 µg mL^−1^.
  i. The six individual synthetic peptides were pooled into a 50 µg mL^−1^ stock, then diluted serially with SPE eluate to 0.5, 1, 2, 5 and 10 µg mL^−1^.
3. The column was preheated to 40 °C and equilibrated with 97% mo mobile phase A and 3% mobile phase B.
  i. Mobile phase A composition: 99.9% water, 0.1% formic acid.
  ii. Mobile phase B composition: 99.9% ACN, 0.1% formic acid.
4. The 5 µg mL^−1^ stock of P1-P6 was initially injected into the column (2 µL) and data collected using the PRM MS method.
  i. This was repeated three times comparing the peak retention times and fragmentation patterns of each sample to ensure instrument and chromatography replicability.
5. A blank sample was injected onto the column (2 µL) and analysed in PRM mode to ensure no carryover was occurring.
6. All samples digested with the INFOGEST protocol and prepared *via* SPE were then analysed using the PRM method.
  i. The injection volume was always 2 µL.
  ii. The P1-P6 standard curve (0.5–10 ppm) was run every 10–15 samples throughout.

**Table 6:** The LOD and LOQ for peptides P1-P6 using the quantitative PRM method described herein.

Peptide	LOQ (µg mL^−1^)	LOD (µg mL^−1^)
P1	0.218	0.072
P2	0.373	0.123
P3	0.258	0.038
P4	0.251	0.027
33mer (P5)	0.312	0.029
P6	0.769	0.161

**Table 7:** The properties of the linear regression modelling of P1-P6 as determined using the least sum of squares method. Pearson's correlation coefficient is displayed.

	Correlation coefficient	y-intercept	Slope	Residual sum of squares
P1	0.99	404,809	3,405,436	1.71 × 10^13^
P2	1.00	224,381	1,776,389	3.49 × 10^13^
P3	1.00	63,340	1,637,587	7.22 × 10^12^
P4	1.00	−13,216	2,063,579	2.60 × 10^13^
P5	0.99	16,042	420,669	7.21 × 10^11^
P6	0.99	−729,751	1,140,396	1.20 × 10^13^

**Table 8:** Day-to-day replicability of the marker peptide concentration in the simulated digesta of bread detected using PRM MS. Samples were analysed each day in triplicate. The mean and CV represent the variation seen between days.

Peptide	Mean concentration (µg mL^−1^)	CV (%)
P1	10.69	5.32
P2	6.23	3.77
P3	2.93	5.52
P4	9.42	4.82
P5	18.38	6.24
P6	4.56	8.64

The raw PRM files were processed using the Xcalibur ‘Quan Browser’ (Thermo Fisher Scientific). Xcalibur integrates the total extracted ion current from the defined fragment ions for each precursor ion, then uses linear regression to calculate the peptide concentration from the standard curve.

1. An automatic processing method (.pmd) was created in the Xcalibur ‘Processing Setup’ tool using the transitions in Table 10.

**Table 10:** Transitions detected during PRM target MS. The HCD fragment ions were used to confirm the identity of each precursor ion during data processing. *L-Phenylalanine-^13^C_9_, ^15^N at residue six.

Peptide name	Amino acid sequence	Molecular weight (kDa)	Precursor m/z (charge state)	Fragment ions m/z	Retention time (min)
P1	LQLQPFPQPQLPY	1.568	784.927 (2+)	279.134 (y2) 483.293 (b4) 470.240 (b6) 1290.719 (b11)	6.11
P1H	LQLQPF*PQPQLPY	1.578	787.927 (2+)	279.134 (y2) 483.293 (b4) 476.254 (b6)* 1296.731 (b11)	6.11
P2	LQLQPFPQPQLPYPQPQPF	2.263	755.068 (3+)	488.251 (y4) 952.526 (b8) 1049.544 (b9)	6.59
P3	LQLQPFPQPQLPYPQPHLPYPQPQPF	3.097	1029.543 (3+)	263.139 (y2) 824.429 (b7) 713.357 (y6) 952.527 (b8) 1290.722 (b11)	7.12
P4	LQLQPFPQPQLPYPQPQLPYPQPQPF	3.087	1032.543 (3+)	263.139 (y2) 488.250 (y4) 713.358 (y6) 973.479 (y8)	6.43
P5 (33mer)	LQLQPFPQPQLPYPQPQLPYPQPQLPYPQPQPF	3.912	978.264 (4+)	488.252 (y4) 824.429 (b21) 973.480 (y8) 1290.726 (b11)	7.29
P6	RPQQPYPQPQPQY	1.626	813.905 (2+)	407.194 (y3) 967.513 (a8) 995.508 (b9)	3.48
2. The .pmd method was assigned as the ‘Proc Method’ in the Xcalibur ‘sequence setup’.
3. Data was processed using the automatic processing function in Xcalibur then viewed in the Quan Browser.
  i. Both the standard curve and peak integration were visually validated.
4. Peptide concentrations were exported to Excel where the SPE dilution factor and P1H suppression were taken into account to produce the final concentration values.

## Conclusion

The protocol described herein allows the user to simulate the digestion of bread (or gluten-containing food) and model the release profile of six immunogenic gluten peptides from within a food matrix. The use of the international standardised INFOGEST *in vitro* digestion protocol increases the relevance of the method to human gastrointestinal digestion and improves the inter-laboratory replicability. The detailed digestion-MS method can compare differences in immunogenic peptide release profile such as those induced by food processing or formulation. The digestion, sample preparation and ionisation parameters described within this manuscript can be applied to a discovery proteomics workflow, to identify and model the release profile of unknown immunogenic gluten peptides. The method development steps highlighted within this manuscript allow future users to incorporate further project-specific gluten peptides.

## Footnotes

1 International Conference on Harmonisation, Validation of Analytical Procedures: Text and Methodology Q2(R1), 2005.
